# Spontaneous murine tumors in the development of patient-derived xenografts: a potential pitfall

**DOI:** 10.18632/oncotarget.27001

**Published:** 2019-06-11

**Authors:** Ann M. Moyer, Jia Yu, Jason P. Sinnwell, Travis J. Dockter, Vera J. Suman, Richard M. Weinshilboum, Judy C. Boughey, Matthew P. Goetz, Daniel W. Visscher, Liewei Wang

**Affiliations:** ^1^ Department of Laboratory Medicine and Pathology, Mayo Clinic, Rochester, MN, USA; ^2^ Department of Molecular Pharmacology and Experimental Therapeutics, Mayo Clinic, Rochester, MN, USA; ^3^ Department of Health Sciences Research, Mayo Clinic, Rochester, MN, USA; ^4^ Department of Surgery, Mayo Clinic, Rochester, MN, USA; ^5^ Department of Oncology, Mayo Clinic, Rochester, MN, USA

**Keywords:** patient-derived xenografts, breast cancer, Ki67, NOD-SCID mice, NSG mice

## Abstract

Patient-derived xenografts (PDX) are generated in immune deficient mice and demonstrate histologic and molecular features similar to their corresponding human tumors. However, murine tumors (non-human) spontaneously occur in these models. 120 consecutive patients with high-risk primary breast cancer enrolled in the prospective neoadjuvant BEAUTY study had tumor tissue obtained at the time of diagnosis. These tumor cells, including initial tissue and subsequent generations, were injected into either NSG (*n* = 365) or NOD-SCID (*n* = 396) female mice. Mice with initial tumor growth sufficient for transfer to the 2nd generation underwent histologic review by pathologists, including Ki67 staining. After passaging the tumors for up to 4 generations, at least one primary mouse tumor was detected from 24 of the 54 PDX-lines, for a frequency of 3.2% (24 mice out of 761 mice), including murine lymphomas (*n* = 13), mammary tumors (*n* = 7), osteosarcomas (*n* = 2), and hemangiosarcomas (*n* = 2). While true PDX showed scattered strong staining with Ki67, murine tumors were Ki67 negative. No significant differences (*p* = 0.062) were observed comparing development of murine tumors in NOD-SCID (*n* = 8) vs NSG mice (*n* = 16). While PDX are a useful tool in cancer research, there is a potential for spontaneous murine tumors to arise, which could alter results of studies utilizing PDX. Morphologic review by a pathologist, potentially along with Ki67 staining, is necessary to ensure that tumor growth represents the desired PDX prior to use in downstream studies. This study is the first prospective study evaluating the frequency, type, and time frame for development of non-human tumors.

## INTRODUCTION

Although significant resources are expended to study tumor biology and develop new therapeutic modalities, it has been estimated that as few as 5–10% of compounds with antineoplastic properties during pre-clinical testing succeed in gaining FDA approval [1, 2]. This is likely in part due to the limitations of model systems that have traditionally been employed in cancer research, including cell lines and animal models. While cancer cell lines were originally derived from patient tumors, they have been substantially manipulated in order to acquire the ability to grow *in vitro*, eliminating the stroma, three-dimensional configuration, and intratumoral cellular heterogeneity. In contrast, although mouse and other animal models of tumors possess stroma and maintain the architecture of a tumor, they are also of limited utility as the biology of the animal and its tumors differ from human biology and human cancer. Patient-derived xenografts (PDX), which are generated by implanting tumor tissue from individual patients into immunocompromised or humanized mice, are a feasible alternative that overcome some of the limitations of traditional model systems in cancer research [3, 4].

PDX are increasingly used in cancer research because they are a renewable source of human tumor that can be used to study the genetics, cellular physiology, and response to therapy of human tumors. PDX show histologic and molecular features similar to the human tumor from which they were derived [5, 6]. Therefore, they are an attractive model for cancer research in that they allow the same “patient” to undergo multiple drug regimens in order to identify the most effective option and to ultimately move toward personalized medicine [7]. Although PDX are a promising model system, their value is limited if they fail to represent the human tumor from which they were derived. Therefore, it is important to carefully characterize each PDX and the parent tumor to ensure similar phenotype and to recognize the differences that may limit the generalizability of findings from experimental work back to patient care. One potential challenge that may be encountered in PDX generation is the spontaneous development of tumors of mouse origin [8, 9]. Although these tumors may show different growth characteristics and are typically histologically distinguishable from human tumors, they represent a potential pitfall that if not identified and excluded, could lead to propagation and use of models that do not represent the patient’s tumor, resulting in erroneous conclusions.

The Breast Cancer Genome Guided Therapy Study (BEAUTY) is a prospective study of patients with high-risk breast cancer treated with neoadjuvant chemotherapy [10]. As part of the BEAUTY study, PDX were derived from both primary percutaneous biopsy specimens, as well as from residual tumor obtained at surgical resection after treatment with neoadjuvant chemotherapy [11]. Here we report on the frequency that spontaneous mouse tumors occurred in female mice in this prospective study, evaluate the type of mouse model from which these tumors were derived (NOD-SCID versus NSG) and the characteristics of these tumors that upon histologic review were found to be of mouse origin rather than the expected PDX, as well as our approach to distinguish these tumors from PDX. This information will aid the diagnostic pathologist and research team in avoiding this potential pitfall.

## RESULTS

**Figure 1:**
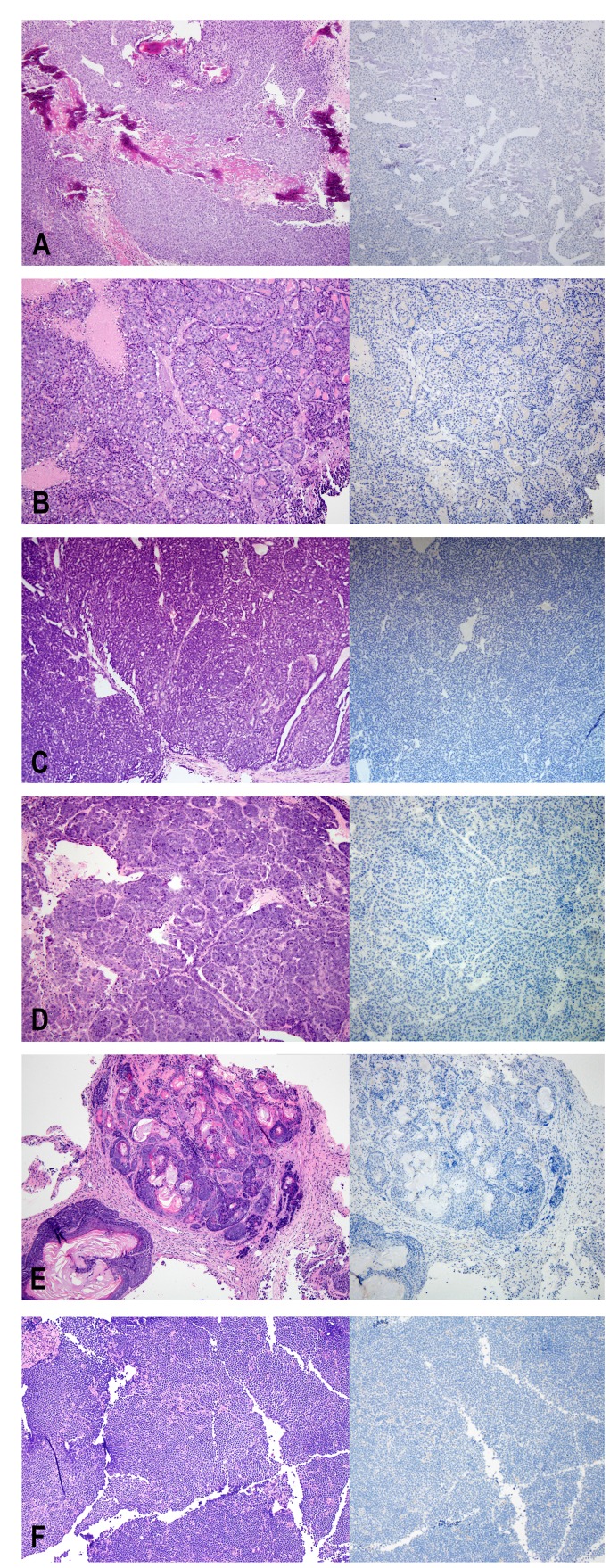
H&E (left) and Ki67 (right) staining of non-human tumors identified at the injection site. (**A**) osteosarcoma, (**B**–**D**) mammary tumors, (**E**) adenoacanthoma, (**F**) lymphoma.

**Figure 2:**
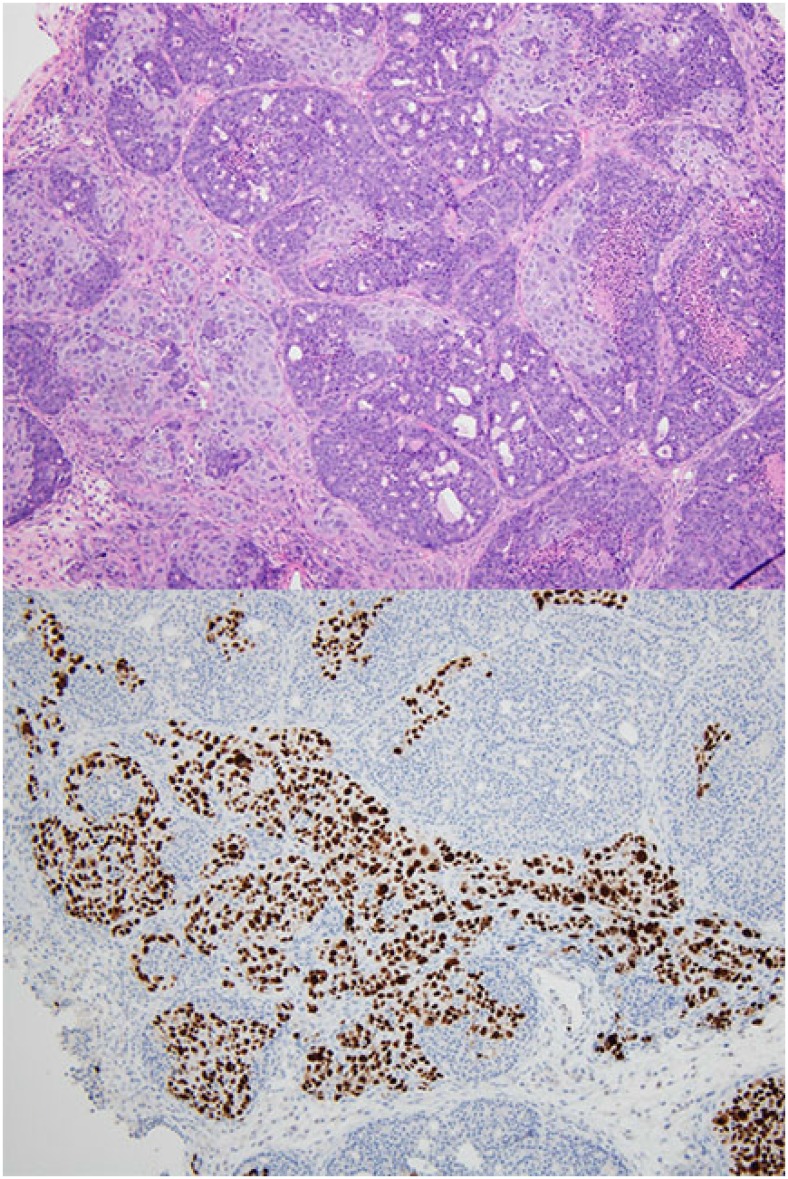
Collision tumor of mouse mammary tumor and patient-derived xenograft. H&E (top), Ki67 stain (bottom). The Ki67 stains only the patient-derived xenograft component and is negative throughout the murine mammary tumor.

From tissue obtained at diagnosis and implanted into female mice, growth at the implantation site was identified in at least one mouse generating 54 PDX lines corresponding to 38 of the 113 evaluable patients enrolled. Data corresponding to 7 patients was not evaluated because tissue was not sent for histologic review. After passaging the tumors for up to 4 generations, at least one primary mouse tumor was detected from 24 of the 54 PDX-lines (Figure 1). Initially, we identified lymphomas, characterized by sheets of uniform and non-cohesive small to medium-sized cells with a high nuclear to cytoplasmic ratio. These malignancies were readily recognized as non-carcinoma, but were not obviously murine on morphology alone. Later, we identified other tumor types, which had morphologic features that were unusual for a PDX derived from human breast carcinoma. These features included markedly smaller cells in some cases as well as metaplastic features such as squamous differentiation, bone formation, and/or an appearance of formation of vascular channels. Tumors with these unexpected features were evaluated in conjunction with review of the International Classification of Rodent Tumors histology text [12] and shared with pathologists in specialized organ system-based working groups for additional confirmation of the diagnostic impression. In total, 13 of the PDX-lines yielded at least one murine lymphoma, 7 at least one murine mammary tumor, 2 murine osteosarcomas, and 2 murine hemangiosarcomas. Among PDX derived from 4 individual patients, more than 1 type of murine tumor grew (lymphoma and mammary tumor in two cases and mammary tumor and hemangiosarcoma in two cases). In addition, PDX derived from 1 patient grew two independent lymphomas. Of the mammary tumors, xenografts from 2 of the PDX-lines were collision tumors including both human and murine cells (Figure 2).

822 total female mice had a primary tumor implanted or a portion of a tumor that was passaged from a prior mouse implanted. Pathology review was performed in batches; therefore, in some instances, a murine tumor was passaged into additional mice prior to learning that the tumor did not correspond to a human xenograft. After excluding these mice, 761 mice remained. Of these 761 female mice, a spontaneous murine tumor developed in 24 (3.2%) (Table 1). Specifically, lymphoma developed in 13 (1.7%), a mammary tumor in 7 (0.9%), osteosarcoma in 2 (0.26%) and hemangiosarcoma in 2 (0.26%). Of the 13 lymphomas, 5 (38.5%) were in NOD-SCID mice and 8 (61.5%) were in NSG mice. Of the mammary tumors, 1 (14.3%) was in a NOD-SCID while 6 (85.7%) were in NSG mice. One NOD-SCID and one NSG mouse developed a hemangiosarcoma and one NOD-SCID and one NSG mouse developed an osteosarcoma. In total, 8 of 396 (2.0%) NOD-SCID mice developed a spontaneous tumor, while 16 of 365 (4.4%) NSG mice developed a spontaneous tumor. This difference was not statistically significant (*p* = 0.062).

**Table 1:** Break-down of non-PDX tumor types identified, along with type of mouse in which tumor was identified, given as count

Tumor type	NOD-SCID	NSG	Total
Lymphoma	5	8	13
Mammary Carcinoma	1	6	7
Hemangiosarcoma	1	1	2
Osteosarcoma	1	1	2
Total	8	16	24

Ki67 is a marker commonly used to measure proliferation. The antibody used by our laboratory is the MIB-1 clone and is a mouse monoclonal antibody that recognizes the human Ki67 antigen. The average percentage of tumor cells staining positive for Ki67 was approximately 50% in the PDX. In contrast, in all of the primary mouse tumors initially identified by morphologic review, Ki67 was negative in all tumor cells, despite expected staining of positive controls run in the same batch (Figure 1). In collision tumors, the Ki67 antibody stained only human nuclei and did not stain nuclei corresponding to the mouse tumor component (Figure 2).

## DISCUSSION

Use of PDX to study tumor biology is becoming more common due to the many advantages of PDX over traditional model systems. Ultimately, it is possible that PDX may become a useful tool to study individual patient tumors and to individualize therapy. However, for this tool to produce accurate results that can be translated to the clinic, great care must be taken to ensure that each PDX model represents the human tumor from which it was derived.

In our study, we generated PDX in order to study high-risk breast cancer. Histologic review of each PDX was performed, primarily to determine how closely each tumor matched the original patient tumor in terms of morphology and immunohistochemical staining patterns for estrogen receptor, progesterone receptor, HER2, and Ki67 [10, 11]. We were surprised to find 24 tumors with unusual morphology and entirely negative staining for Ki67. When these tumors were further passaged to additional mice, they continued to grow. After careful examination, on the basis of morphology, we diagnosed these unusual tumors as primary mouse tumors [12].

Lymphomas, particularly of thymic origin, have previously been reported to occur in at high frequency in NOD-SCID mice, resulting in a mean lifespan of 8.5 months [8, 13, 14]. In contrast, NSG mice are expected to be resistant to these tumors and typically survive for over 16 months [15, 16]. However, in an early study characterizing NSG mice, out of the 34 mice studied, five appeared moribund between the ages of 47 to 68 weeks and at necropsy two were found to have lymphoma (non-thymic), one had mammary adenocarcinoma, and for two the cause of death was undetermined [15]. In our study, we identified 5 NOD-SCID mice and 8 NSG mice that developed spontaneous lymphoma, though we did not fully characterize the type of lymphoma. A recent report by Zhang et al. suggested that a high percentage of PDX in NOD-SCID mice demonstrated human-derived lymphoma of a B-cell subtype positive for Epstein-Barr virus [17]. Our Ki67 stain, which is specific for proliferating human cells, was negative in the 13 lymphomas we observed, indicating that they were of mouse origin, rather than human origin.

In addition to lymphomas, we observed mammary carcinomas in several mice. Morphologically, mouse mammary carcinomas differed from PDX. Grossly, mammary gland tumors in the mouse can be identified as subcutaneous nodules present anywhere from the chin to the pelvic region and on the dorsal, lateral, or ventral surface [18]. This wide variation in location at presentation may make them difficult to distinguish from the injected human tumor. Most of the murine mammary carcinomas showed a proliferation of small cuboidal cells arranged in acinar and tubular growth patterns. Necrosis was frequently identified. Many of the primary mouse tumors also showed areas of squamous differentiation with abrupt transition from glandular neoplastic cells to cells forming mature keratin—a feature not regularly identified in human breast cancer with squamous differentiation. Some of these tumors had sufficient squamous metaplasia to be classified as adenoacanthomas. In collision tumors containing both cells consistent with PDX and primary murine mammary carcinoma, the tumor cells of mouse origin tended to be smaller and had relatively more condensed chromatin. In addition, the mouse cells were universally negative for Ki67, while many of the cells of human origin stained strongly positive.

In our population of NSG and NOD-SCID female mice we also observed several spontaneous osteosarcomas and hemangiosarcomas. These are not well characterized in the literature; however, both tumor types have previously been reported to spontaneously develop in laboratory mice [12, 18–21].

A previous longitudinal survival study of female NSG breeding mice that were allowed to live their natural lifespan found a variety of inflammatory and neoplastic conditions that contributed to morbidity and mortality [22]. Our study, which also included female mice, differed in that the mice were administered exogenous estrogen and a core of human breast tumor tissue was implanted. Lymphomas developed within a few weeks of injection of the human tissue in younger mice, while the other tumor types were identified in older mice, similar to the tumors in the longitudinal study, and long after the initial injection. While we observed tumor growth at the injection site, in all except the two cases involving collision tumors, there was no evidence of successful PDX growth in the mice developing a spontaneous tumor. Multiple masses were identified in some lymphoma cases; otherwise, we did not observe any spontaneous tumors away from the implantation site. However, due to the nature of the study focusing on generation of PDX, organs that appeared grossly normal were not thoroughly sampled, so the possibility of small tumors away from the injection site cannot be excluded. The observation that the spontaneous tumors were identified at the site of injection of human tumor tissue raises the possibility that the tumor tissue may alter the local microenvironment to predispose the mouse to tumorigenesis; however, based on our data, no definitive conclusions can be drawn and further investigation is required to explore this possibility.

Contamination of cell lines is a well-recognized and long standing problem that can lead to results that do not reflect the biology of the tumor type of interest and has resulted in many journals requiring verification of cell lines prior to publication [23]. Similarly, validation of PDX will be important to ensure that the tumor is representative so that the results are clinically relevant. Evaluation by a pathologist is one approach to validate that the PDX is of the appropriate tumor type. However, most pathologists are not routinely exposed to veterinary pathology during the course of their training. Therefore, it is essential for pathologists who are performing xenograft review to be aware of the possibility of spontaneous mouse tumors and to have some familiarity with their appearance to avoid spuriously characterizing a mouse tumor as a legitimate PDX. In addition, in our experience, immunohistochemical staining with the Ki67 MIB-1 clone serves as a robust and reliable marker to differentiate between human and mouse tumors in cases where the morphologic features may be ambiguous.

Patient-derived xenografts are a promising tool to enhance cancer research and personalized therapy. However, as we have demonstrated, care must be taken to ensure that the mass that grows after injection of patient tumor is of human origin. Histologic review is one method for validation, and it is essential for pathologists reviewing PDX to be aware of the possibility of spontaneous mouse tumors. We have demonstrated that Ki67 is a useful adjunct to histologic evaluation, which may be particularly helpful for pathologists unfamiliar with murine tumors and in difficult cases where the morphologic features do not readily allow for distinction. These results also highlight the necessity to validate PDX prior to subjecting them to experiments and potentially guiding patient care.

## MATERIALS AND METHODS

### Subjects

Of the 140 patients enrolled in the BEAUTY STUDY, 120 patients at either Mayo Clinic Rochester (Minnesota) or Florida were enrolled in the PDX portion of the study and had at least one tumor biopsy submitted for mouse injection [10, 11]. Initial percutaneous needle core biopsies of the primary breast tumor were obtained at the time of diagnosis prior to neoadjuvant chemotherapy. This study was approved by the Mayo Clinic Institutional Review Board.

### Generation of patient-derived xenografts

Patient-derived xenografts were generated as described previously [11]. Specifically, sterile fresh tumor was kept on ice in sterile phosphate-buffered saline (PBS) for implantation and a portion of the tumor was formalin-fixed and paraffin-embedded for analysis. Tumor tissue was received in the laboratory within an hour of biopsy. 6–8 week old female non-obese diabetic/severe combined immunodeficient (NOD-SCID) or NOD-SCID/IL2γ-receptor null (NSG) mice purchased from the Jackson Laboratories (Bar Harbor, Maine) were pre-treated with 0.16μg/mL 17β-estradiol (Sigma-Aldrich, St. Louis, MO) in water for at least one week prior to tumor implantation and continued on low dose estrogen supplementation throughout the study. Fragments of approximately 4 mm^3^ were injected subcutaneously into the mice along with growth factor reduced Matrigel (BD Biosciences, San Jose, CA) using a 14 gauge trocar. Mice were palpated weekly and digital calipers were used to measure tumor growth. When the tumors reached 200–1500 mm^3^, mice were sacrificed. After harvesting the tumor, a portion was fragmented and transplanted into additional mice, a portion was formalin-fixed and paraffin embedded for histologic analysis, and portions were frozen for additional analyses and future engraftment. All mouse experiments were reviewed and approved by the Mayo Clinic Institutional Animal Care and Use Committee.

### Immunohistochemical staining

All tumor samples were fixed for 6–72 hours in 10% neutral buffered formalin within 1 hour of resection, followed by paraffin embedding. Tumor morphology was evaluated on slides stained with hematoxylin and eosin (H&E). Tumors were also evaluated for the presence or complete absence of immunohistochemical staining with a monoclonal antibody against Ki67 (MIB-1 clone, Dako, Carpinteria, CA), which was performed in the Mayo Clinic Pathology Research Core.

### Statistical analysis

The percentage of NOD-SCID and NSG female mice that developed a spontaneous murine tumor was compared by the chi-square test of equal proportions.
